# Validation of the novel GLAS algorithm as an aid in the detection of liver fibrosis and cirrhosis based on GP73, LG2m, age, and sex

**DOI:** 10.1186/s12014-023-09444-7

**Published:** 2023-11-28

**Authors:** Philip M. Hemken, Xuzhen Qin, Lori J. Sokoll, Laurel Jackson, Fan Feng, Peng Li, Susan H. Gawel, Bailin Tu, Zhihong Lin, James Hartnett, David Hawksworth, Bryan C. Tieman, Toru Yoshimura, Hideki Kinukawa, Shaohua Ning, Enfu Liu, Fanju Meng, Fei Chen, Juru Miao, Xuan Mi, Xin Tong, Daniel W. Chan, Gerard J. Davis

**Affiliations:** 1 Diagnostics Discovery Research & Development, Abbott Diagnostics, 100 Abbott Park Road AP20, Abbott Park, IL 60064 USA; 2 Department of Laboratory Medicine, Chinese Academy of Medical Sciences & Peking Union Medical College Hospital, Beijing, China; 3 Division of Clinical Chemistry, Department of Pathology, The Johns Hopkins University, Baltimore, MD USA; 4 Research & Development, Abbott Japan, Matsudo, Japan; 5 Diagnostics Discovery Research & Development, Abbott Diagnostics, Shanghai, China

**Keywords:** Hepatitis, Risk assessment, Diagnostic, Liver Disease, Immunoassay, Predictive model

## Abstract

**Background:**

Diagnosis of liver disease at earlier stages can improve outcomes and reduce the risk of progression to malignancy. Liver biopsy is the gold standard for diagnosis of liver disease, but is invasive and sample acquisition errors are common. Serum biomarkers for liver function and fibrosis, combined with patient factors, may allow for noninvasive detection of liver disease. In this pilot study, we tested and validated the performance of an algorithm that combines GP73 and LG2m serum biomarkers with age and sex (GLAS) to differentiate between patients with liver disease and healthy individuals in two independent cohorts.

**Methods:**

To develop the algorithm, prototype immunoassays were used to measure GP73 and LG2m in residual serum samples collected between 2003 and 2016 from patients with staged fibrosis and cirrhosis of viral or non-viral etiology (*n* = 260) and healthy subjects (*n* = 133). The performance of five predictive models using combinations of age, sex, GP73, and/or LG2m from the development cohort were tested. Residual samples from a separate cohort with liver disease (fibrosis, cirrhosis, or chronic liver disease; *n* = 395) and healthy subjects (*n* = 106) were used to validate the best performing model.

**Results:**

GP73 and LG2m concentrations were higher in patients with liver disease than healthy controls and higher in those with cirrhosis than fibrosis in both the development and validation cohorts. The best performing model included both GP73 and LG2m plus age and sex (GLAS algorithm), which had an AUC of 0.92 (95% CI: 0.90–0.95), a sensitivity of 88.8%, and a specificity of 75.9%. In the validation cohort, the GLAS algorithm had an estimated an AUC of 0.93 (95% CI: 0.90–0.95), a sensitivity of 91.1%, and a specificity of 80.2%. In both cohorts, the GLAS algorithm had high predictive probability for distinguishing between patients with liver disease versus healthy controls.

**Conclusions:**

GP73 and LG2m serum biomarkers, when combined with age and sex (GLAS algorithm), showed high sensitivity and specificity for detection of liver disease in two independent cohorts. The GLAS algorithm will need to be validated and refined in larger cohorts and tested in longitudinal studies for differentiating between stable versus advancing liver disease over time.

**Supplementary Information:**

The online version contains supplementary material available at 10.1186/s12014-023-09444-7.

## Background

Liver disease is a major cause of death in many countries [1], and patients with liver fibrosis and cirrhosis are at high risk of developing liver cancer. Liver cancer is ranked among the top three cancers in 46 countries. In 2020, 905,700 persons worldwide were diagnosed with liver cancer, and 830,200 died from the disease. The global burden of primary liver cancer is estimated to increase by more than 55% from 2020 to 2040 [1]. Earlier diagnosis of liver disease can improve outcomes and reduce the risk of progression to malignancy.

Current clinical practice guidelines include liver biopsy as the gold standard for the diagnosis of liver fibrosis and cirrhosis [2]; however, liver biopsy is invasive and is prone to sampling error. Noninvasive tests of liver function and fibrosis provide additional diagnostic information, and diagnostic accuracy can be improved by combining information into algorithms that include patient factors and clinical biomarkers. Current diagnostic algorithms include Fib-4, the enhanced liver fibrosis (ELF) test, the aspartate platelet ratio index (APRI), Fibrotest (CE)/FibroSure (US), and Fibrometer (see Additional File 1). Imaging tests include Fibroscan and magnetic resonance elastography (MRE). While these tests and algorithms help guide clinical decision making, each of them has drawbacks and limitations. Additional biomarkers are needed to improve the accuracy of current methods, particularly for the earlier detection of liver fibrosis and cirrhosis.

GP73 was initially described as a novel Golgi-localized protein that is upregulated in viral and nonviral liver disease [3]. A subsequent study found that GP73 protein levels were minimal or undetected in normal liver but significantly elevated in hepatitis B virus (HBV)-, hepatitis C virus (HCV)-, and alcohol-induced liver disease, as well as autoimmune liver disease [4]. Several groups have since developed immunoassays to study serum GP73 levels for the diagnosis of liver disease [5–14] including chronic hepatitis, cirrhosis, and hepatocellular carcinoma (HCC). One study found that GP73 detected by enzyme-linked immunosorbent assay (ELISA) was a more accurate biomarker of liver fibrosis compared to Fib-4 (area under the curve [AUC] 0.751 for Fib-4 versus 0.898 for GP73) [14]. However, we previously showed that GP73 was not significantly elevated in HCC compared to the biomarker PIVKA-II (protein induced by the absence of vitamin K or antagonist-II) and AFP [15].

Laminin-332 (Ln-322) is abundant in HCC tissue, where it has been reported to support proliferation, migration, and invasion of tumor cells [16–19]. More specifically, a component of Ln-322, the Laminin-gamma 2 monomer (LG2m), is frequently expressed in several types of malignant cancer cells and tissues [20] and promotes the adhesion, migration, and scattering of HCC cells [21]. LG2m may play a crucial role in cancer invasion and metastasis as it is deposited in high concentrations at the invading edge of solid tumors, where it mediates the migration and invasion of transformed cells [20, 22, 23]. A previous study reported that serum LG2m levels were significantly elevated in patients with HCC and the presence of both LG2m and PIVKA-II was more sensitive for diagnosis of HCC than existing liver tumor markers [24]. In addition, the level of LG2m was found to predict extrahepatic spread in patients with HCC and the development of HCC in patients with chronic hepatitis C who achieved a sustained virological response [25]. Therefore, LG2m in human serum may be useful as a biomarker for HCC surveillance and risk stratification for HCC development and metastasis in patients with chronic liver disease.

In previous work, we developed and validated a new algorithm for the early detection of HCC that included age, sex, alpha fetoprotein (AFP), and PIVKA-II (ASAP) [26]. Other studies have since validated the same four biomarkers using different statistical approaches (e.g., GAAD/GALAD and GAAP models) [27, 28] and have developed an online calculator for detecting HCC in patients with HBV [29]. Applying the same approach here, we conducted a pilot study to evaluate the utility of combining GP73, LG2m, age, and sex (GLAS algorithm) for the detection of liver fibrosis and cirrhosis. GP73 and LG2m were measured using newly developed, robust chemiluminescent immunoassays run on the Abbott ARCHITECT system. A pilot study of the GLAS algorithm was performed at Johns Hopkins University School of Medicine (JHU) and a validation study was performed at Peking Union Medical College Hospital (PUMCH) in China.

## Methods

### GP73 antibodies and immunoassay development

Mice were immunized with recombinant GP73 and fusions were performed with an NSO myeloma cell line to produce monoclonal antibodies. Twelve IgG antibodies were produced in-house and were screened for use in a prototype GP73 ARCHITECT immunoassay, for a total of approximately 144 antibody pairs. The best antibody pairs were initially selected based on sensitivity, range, and reagent stability. The capture IgG1 (kappa) antibody was coated on magnetic microparticles. The conjugate antibody was murine human chimeric Fab (muhuFab) produced in CHO cells. This conjugate design minimized interference by human anti-mouse antibodies (HAMA) because it lacks the Fc region of the antibody and provided better sensitivity [30]. Microparticle reagent bulk stability was tested under heat stress for 3 days at 45 °C compared to controls at 2–8 °C (see Additional File 2). The conjugate reagent bulk stability was tested with heat stress at 30 and 37 °C for 7 and 14 days compared to the 2–8 °C control condition (see Additional File 3). Further prototype verification studies with the selected antibody pair included reagent stability, limit of blank, limit of detection, and limit of quantitation (LoBDQ), dilution linearity, 20-day precision, range, and interference testing.

### LG2m assay development

A hybridoma of an anti-LG2m monoclonal antibody (Clone 1) used for capture was originally developed by Koshikawa et al. [31]. A hybridoma of an anti-LG2m monoclonal antibody (Clone 2) used for detection was developed by Abbott Laboratories (Lake County, IL, USA). Clone 1 detected only the LG2m monomer, and not LG2m as a component of Ln-332. Monoclonal antibodies were prepared and purified by Abbott Laboratories on a protein A column. The capture antibody (Clone 1) was produced in CHO cells and coated on magnetic microparticles. The conjugate antibody (Clone 2) was labeled with acridinium for the detection of LG2m. Assay prototype verification studies including LoBDQ, dilution linearity, 20-day precision, range, auto-dilution, and interference testing were performed.

### Study design and serum samples

The prototype GP73 and LG2m ARCHITECT immunoassays were used to measure GP73 and LG2m concentrations in residual serum samples collected between 2003 and 2016 at JHU in Baltimore, MD, from patients with fibrosis or cirrhosis with viral or non-viral etiology, and healthy controls (n = 147) [26]. All fibrosis samples had a known stage determined by traditional methods of biopsy and standard liver enzyme tests. Additional residual serum samples were analyzed at JHU that had been collected after obtaining informed consent from patients with liver cirrhosis at the University of Texas Southwestern Medical Center (UTSMC) in Dallas, TX. For each residual serum sample, the following de-identified data was collected: age, sex, race/ethnicity, and etiology of liver disease. An additional set of serum samples (collectively referred to as the Western Vendor Cohort, WVC; *n* = 246) were purchased from BioIVT (Wesbury, NY), Biomex GmbH (Heidelberg, Germany), Discovery Life Sciences (Huntsville, AL), and ProMedDx (Norton, MA). These three sample sets (JHU/UTSMC/WVC) were combined to develop and train the liver fibrosis and cirrhosis diagnostic algorithm (development cohort). The study was approved by the JHU IRB (#IRB00196747).

For the validation cohort, samples were obtained from PUMCH (Bejing, China), from patients with liver disease (fibrosis, cirrhosis, or chronic liver disease) and healthy subjects (*n* = 501). This cohort was used to validate the model derived from the development cohort. Patients with chronic liver disease included those with fatty liver disease, HBV-induced liver disease, and/or autoimmune hepatitis. For each serum sample, the following de-identified data was collected: age, sex, race/ethnicity, and etiology of liver disease. The study was approved by the PUMCH IRB (HS-2386).

### Sample storage and assays

Serum samples were stored at approximately − 80 °C prior to analysis. GP73 and LG2m levels were measured using the prototype GP73 and LG2m ARCHITECT immunoassays on an ARCHITECT *i*2000SR analyzer (Abbott Laboratories, North Chicago, IL). Each two-step sandwich immunoassay utilizes paramagnetic microparticles coated with either anti-GP73 [32] or anti-LG2m [33] antibodies and produces a chemiluminescent signal for the quantitative measurement of GP73 or LG2m in human serum and plasma. The performance characteristics for the prototype ARCHITECT GP73 and LG2m assays are described in Table 1. Both assays were analytically robust, with performance similar to that of other automated in vitro diagnostic immunoassays.

**Table 1:** Performance characteristics of the prototype GP73 and LG2m assays

Parameter	GP73 ARCHITECT Assay	LG2m Alinity i Assay
20-day precision	Total within-laboratory %CV of less than 3.0%	Total within-laboratory %CV of less than 4.6%
LoQ	0.20 ng/mL	3.45 pg/mL
LoD	0.04 ng/mL	0.13 pg/mL
Dilution linearity	Deviation from linearity (DL) of < 10% within the range of 10 ng/mL to 1,000 ng/mL	Deviation from linearity (DL) within ± 3 pg/mL for sample < 30 pg/mL, ± 10% for samples 30 to 5,000 pg/mL
Measuring interval	LoQ – 1000 ng/mL	LoQ – 50,000 pg/mL
Extended range with autodilution	n/a	1:10 autodilution to 50,000 pg/mL
HAMA/RF and interferences	Within ± 10% for HAMA/RF and potential interferents, no notable endogenous interferences observed	Within ± 10% for HAMA/RF* and potential interferents, no notable endogenous interferences observed

CV, coefficient of variation; HAMA, human anti-mouse antibodies; LoD, limit of detection; LoQ, limit of quantitation; RF, rheumatoid factor

*The LG2m assay showed differences in measured concentration of LG2m within ± 10% for samples when spiked with HAMA at a concentration of 900 ng/mL and purified RF at a concentration of 600 IU/mL.

### Statistical analysis

Biomarker concentrations were stratified by disease category. The probability of each biomarker to detect non-cancer liver disease (chronic liver disease, fibrosis, and/or cirrhosis) was determined and logistic regression (LR) classification models were used to explore the best combination of biomarkers for the detection of non-cancer liver disease. For biomarkers with skewed distribution, logarithmic transformation was applied prior to modeling. Wilcoxon tests were used for significance testing.

All of the samples from the development cohort (JHU/UTMSC/WVC) were used to train the models. The response variable for the models was the binary liver disease status (fibrosis and/or cirrhosis versus healthy). Multiple LR models were developed by selecting different combinations of age, sex, and the two biomarkers as the classifiers. The best model was selected based on the combination of classifiers with the highest ROC AUC. Confidence intervals for AUCs were calculated by taking 2000 stratified bootstrapped replicates. The sensitivities (SEs) and specificities (SPs) were reported at the default cutoff of 0.5 for LR. Additionally, sensitivity at a fixed specificity of 90% was reported as the median value across 2000 stratified bootstrapped replicates. Median values were also calculated from specificity at fixed sensitivities of 90% and 75%. AUCs were compared by pairing AUC curves as described by Delong et al. [34].

The best model selected from the development cohort was further assessed. To evaluate the generalizability of the best model in a different population, an independent validation cohort was used to validate model performance. Non-cancer liver disease was added to chronic liver disease (including fatty liver disease, HBV-induced liver disease, and/or autoimmune hepatitis) to better evaluate the performance in clinical practice.

All statistical analyses were performed using R 4.1.1 (The R Foundation for Statistical Computing).

## Results

### Algorithm development cohort demographics

The development cohort consisted of serum samples from 78 patients with fibrosis (with or without hepatitis), 182 patients with cirrhosis (with or without hepatitis), and 133 healthy subjects (Table 2; *N* = 393). The median ages for patients in the fibrosis, cirrhosis, and healthy control groups were 54 (interquartile range [IQR] 45–63), 56 (IQR 48–63), and 40 (IQR 33–56) years, respectively, with the majority of patients being White males (fibrosis/cirrhosis) or Black males (healthy).

**Table 2:** Development Cohort Demographics (*N* = 393)

Characteristic	JHU/UTSMC (*N* = 147)			WVC (*N* = 246)			Overall (*N* = 393)		
	**Healthy** (*n* = 34)	**Fibrosis** (*n* = 19)	**Cirrhosis** (*n* = 94)	**Healthy** (*n* = 99)	**Fibrosis** (*n* = 59)	**Cirrhosis** (*n* = 88)	**Healthy** (*n* = 133)	**Fibrosis** (*n* = 78)	**Cirrhosis** (*n* = 182)
**Age**									
Median (IQR)	60 (51, 65)	47 (43, 51)	56 (49, 61)	37 (29, 46)	57 (49, 67)	54 (48, 65)	40 (33, 56)	54 (45, 63)	56 (48, 63)
Range	40, 77	31, 57	23, 73	21, 65	20, 81	26, 94	21, 77	20, 81	23, 94
**Sex**, *n* (%)									
Male	17 (50%)	16 (84%)	56 (60%)	79 (80%)	27 (46%)	51 (58%)	96 (72%)	43 (55%)	107 (59%)
Female	17 (50%)	3 (16%)	38 (40%)	20 (20%)	32 (54%)	37 (42%)	37 (28%)	35 (45%)	75 (41%)
**Race**, *n* (%)									
White	22 (67%)	10 (53%)	53 (58%)	5 (5.1%)	51 (88%)	43 (90%)	27 (20%)	61 (79%)	96 (69%)
Black	1 (3.0%)	5 (26%)	30 (33%)	94 (95%)	2 3.4%)	1 (2.1%)	95 (72%)	7 (9.1%)	31 (22%)
Hispanic	0 (0%)	2 (11%)	1 (1.1%)	0 (0%)	4 (6.9%)	2 (4.2%)	0 (0%)	6 (7.8%)	3 (2.1%)
Asian	0 (0%)	1 (5.3%)	3 (3.3%)	0 (0%)	0 (0%)	0 (0%)	0 (0%)	1 (1.3%)	3 (2.1%)
Other/Mixed Race	10 (30%)	1 (5.3%)	5 (5.4%)	0 (0%)	1 (1.7%)	2 (4.2%)	10 (7.6%)	2 (2.6%)	7 (5.0%)
Unknown	1	0	2	0	1	40	1	1	42
**Etiology**, *n* (%)	—			—			—		
** Viral**									
HBV		0 (0%)	13 (14%)		0 (0%)	20 (23%)		0 (0%)	33 (18%)
HCV		0 (0%)	41 (44%)		0 (0%)	22 (25%)		0 (0%)	63 (35%)
** Non-Viral**									
Alcoholic Liver Disease		0 (0%)	0 (0%)		8 (14%)	0 (0%)		8 (10%)	0 (0%)
Fatty Liver Disease		0 (0%)	0 (0%)		33 (56%)	0 (0%)		33 (42%)	0 (0%)
Hemochromatosis		0 (0%)	0 (0%)		8 (14%)	0 (0%)		8 (10%)	0 (0%)
Simple Steatosis		0 (0%)	0 (0%)		6 (10%)	0 (0%)		6 (7.7%)	0 (0%)
Non-Viral Cirrhosis		0 (0%)	40 (43%)		0 (0%)	0 (0%)		0 (0%)	40 (22%)
** Unknown**		19 (100%)	0 (0%)		4 (6.8%)	46 (52%)		23 (29%)	46 (25%)

JHU, Johns Hopkins University School of Medicine; UTSMC, University of Texas Southwestern Medical Center; WVC, Western Vendor Cohort; IQR, interquartile range; HBV, hepatitis B virus; HCV, hepatitis C virus

### Algorithm validation cohort demographics

The validation cohort included 503 individuals; of these, 501 were included in this analysis and 2 were excluded for missing assay results as these specimens had been depleted prior to the study. The validation cohort included 119 patients with cirrhosis, 129 patients with fibrosis, 147 patients with chronic liver disease, and 106 healthy subjects (Table 3). Patient age ranged from 19 to 88 years, with a greater proportion of men in the chronic liver disease and fibrosis groups and a greater proportion of women in the healthy and cirrhosis groups. All individuals in the validation cohort were Asian.

**Table 3:** Validation Cohort Demographics (*n* = 501)

Characteristic	Healthy *N* = 106	CLD *N* = 147	Fibrosis *N* = 129	Cirrhosis *N* = 119
**Age**				
Median (IQR)	36 (30, 42)	43 (35, 51)	55 (42, 67)	58 (52, 66)
Range	21, 71	23, 78	19, 88	32, 86
**Sex**, *n* (%)				
Male	30 (28%)	80 (54%)	69 (53%)	30 (25%)
Female	76 (72%)	67 (46%)	60 (47%)	89 (75%)
**Race**, *n* (%)				
Asian	106 (100%)	147 (100%)	129 (100%)	119 (100%)
**Etiology**, *n* (%)	—			
**Viral**				
HBV		89 (61%)	30 (23%)	17 (14%)
HCV		0 (0%)	2 (1.6%)	3 (2.5%)
Fatty Liver Disease & HBV		6 (4.1%)	0 (0%)	0 (0%)
**Non-Viral**				
Alcoholic Liver Disease		0 (0%)	4 (3.1%)	0 (0%)
Autoimmune Hepatitis		6 (4.1%)	2 (1.6%)	0 (0%)
Fatty Liver Disease		45 (31%)	4 (3.1%)	2 (1.7%)
Fatty Liver Disease & Autoimmune Hepatitis		1 (0.7%)	0 (0%)	0 (0%)
Primary Biliary Cirrhosis		0 (0%)	0 (0%)	81 (68%)
Non-Viral Cirrhosis		0 (0%)	0 (0%)	9 (7.6%)
Non-Viral Fibrosis		0 (0%)	44 (34%)	0 (0%)
** Unknown**		0 (0%)	43 (33%)	7 (5.9%)

CLD, chronic liver disease; IQR, interquartile range; HBV, hepatitis B virus; HCV, hepatitis C virus

### Biomarker concentrations

**Fig. 1:**
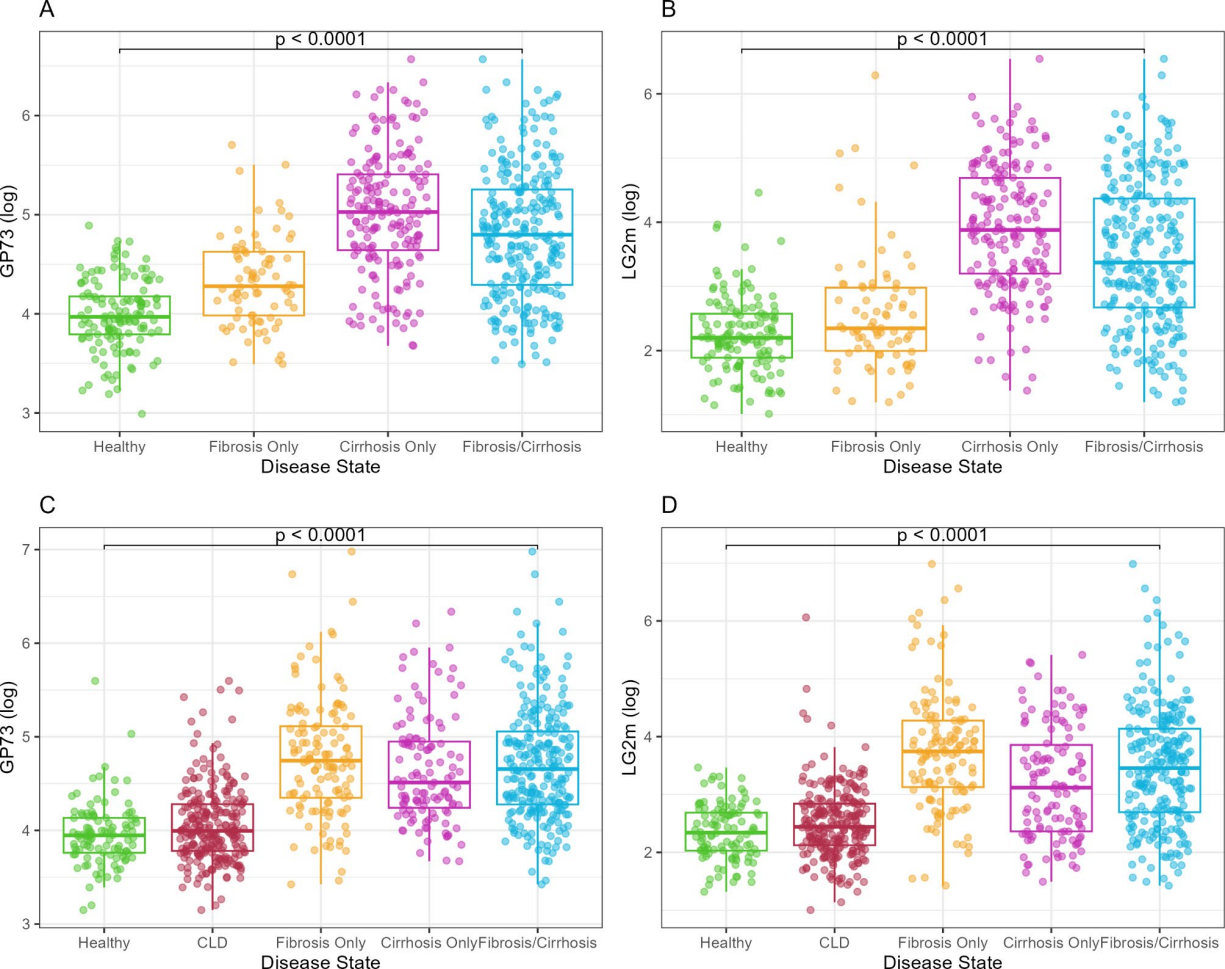
Distribution of GP73 and LG2m by disease state in the development **(A, B)** and validation cohorts **(C, D)**. Biomarker concentrations were significantly different between healthy and disease (fibrosis/cirrhosis) samples (Wilcoxon test)

In the development cohort, median GP73 and LG2m concentrations were found to be higher in patients with fibrosis/cirrhosis (GP73 121.27 ng/mL; LG2m 29.16 pg/mL) than in healthy controls (GP73 52.99 ng/mL; LG2m 9.01 pg/mL). On average, patients with cirrhosis had higher median biomarker concentrations (GP73 152.49 ng/mL; LG2m 48.39 pg/mL) compared to patients with fibrosis GP73 72.12 ng/mL; LG2m 10.46 pg/mL; Fig. 1A, B). In the validation cohort, median GP73 and LG2m levels were also higher overall in patients with fibrosis/cirrhosis (GP73 105.14 ng/mL; LG2m 31.70 pg/mL) compared to healthy controls (GP73 51.80 ng/mL; LG2m 10.40 pg/mL; Fig. 1C, D). However, both GP73 and LG2m median concentrations in the validation cohort were slightly higher for patients with fibrosis (GP73 115.10 ng/mL; LG2m 42.30 pg/mL) compared to patients with cirrhosis (GP73 91.11 ng/mL; LG2m 22.65 pg/mL; Fig. 1C, D). On average, biomarker levels for patients with chronic liver disease fell between those in the fibrosis/cirrhosis and healthy groups, with a median GP73 concentration in the chronic liver disease group of 57.76 ng/mL and 12.75 pg/mL for LG2m (Fig. 1C, D).

### Model performance in the development cohort

Five models were created and compared from the development cohort data using four potential variables: age, sex, GP73, and/or LG2m (Table 4). The AUC for differentiating fibrosis/cirrhosis from healthy controls was slightly higher for GP73 alone (Model 1: 0.86, 95% CI: 0.82–0.89) compared to LG2m alone (Model 2: 0.83, 95% CI: 0.79–0.87), but the difference was not statistically significant. The addition of age and sex to either the GP73 or LG2m models increased AUCs (Model 3: 0.91, 95% CI: 0.89–0.94 and Model 4: 0.88, 95% CI: 0.85–0.92, respectively). The AUC values from both updated models were improved and statistically significant compared to the individual biomarkers alone (*p* < 0.0001 and *p* = 0.0003, respectively).

**Table 4:** Diagnostic Performance of Biomarkers Alone and in Combination with Clinical Factors in the Development Cohort (JHU/UTSMC/WVC) and Model 5 in the Validation Cohort (PUMCH)

	**Model**	**Predictor Variables**	**N**	**Events (** **n** **)**	**AUC**	**AUC 95% CI**	**SE (%)**	**SP (%)**	**SE (SP = 90%)**	**SP (SE = 90%)**	**SP (SE = 75%)**
Development Cohort	1	GP73	393	260	0.86	(0.82, 0.89)	82.7	64.7	68.8	47.4	78.9
	2	LG2m	393	260	0.83	(0.79, 0.87)	81.9	60.9	66.5	32.3	79.7
	3	GP73 + Age + Sex	393	260	0.91	(0.89, 0.94)	89.6	73.7	76.2	73.7	91.7
	4	LG2m + Age + Sex	393	260	0.88	(0.85, 0.92)	88.5	75.9	61.9	72.9	84.2
	5	GP73 + LG2m + Age + Sex (GLAS)	393	260	0.92	(0.90, 0.95)	88.8	75.9	79.2	73.7	94.7
	**Model**	**Etiology**	***N***	**Predicted Events (** *n* **)**	**AUC**	**AUC 95% CI**	**SE (%)**	**SP (%)**	**SE (SP = 90%)**	**SP (SE = 90%)**	**SP (SE = 75%)**
Validation Cohort	5 (GLAS)*	All Fibrosis/Cirrhosis	354	248	0.93	(0.90, 0.95)	91.1	80.2	81.0	82.1	93.4
		Viral	158	52	0.91	(0.86, 0.96)	88.5	80.2	75.0	81.1	91.5
		Non-viral	252	146	0.94	(0.91, 0.97)	93.2	80.2	86.3	84.9	93.4
		Unknown Etiology	156	50	0.91	(0.86, 0.96)	88.0	80.2	74.0	73.6	89.6
		Chronic Liver Disease	253	147	0.65	(0.58, 0.71)	42.9	80.2	26.5	15.1	39.6
		All Liver Disease	501	395	0.82	(0.78, 0.86)	73.2	80.2	61.0	36.8	76.4

AUC, area under the ROC curve; SE, sensitivity; SP, specificity

*Comparison group for all analyses is healthy controls (*n* = 106)

**Fig. 2:**
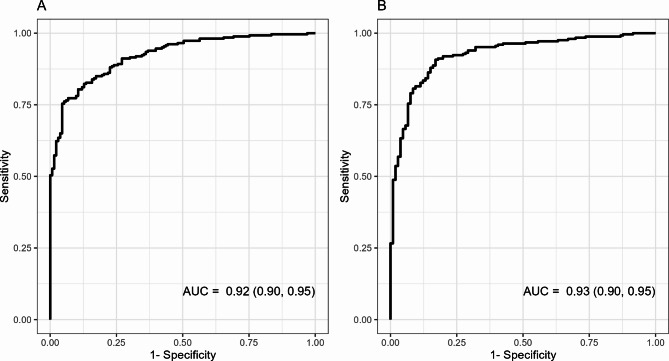
ROC curves for the GLAS algorithm (Model 5) in the development **(A)** and validation **(B)** cohorts for fibrosis/cirrhosis versus healthy subjects

The best model included all four variables, GP73, LG2m, age, and sex (the GLAS algorithm), and increased the AUC to 0.92 (Model 5: 95% CI: 0.90–0.95), with a sensitivity of 88.8% and a specificity of 75.9% (Fig. 2A). The increase was statistically significant compared to GP73 or LG2m alone (Models 1 and 2) and the model with LG2m, age, and sex (Model 4; all *p*-values < 0.0001). The increase was not statistically different compared to Model 3 with GP73, age, and sex (*p* = 0.0621).

### Model validation in an independent cohort

The best model from the development cohort (Model 5, the GLAS algorithm) was evaluated using the validation cohort data as an independent assessment of clinical performance (Table 4). The GLAS algorithm had an estimated AUC for fibrosis/cirrhosis of 0.93 (95% CI: 0.90–0.95) in the validation cohort (Fig. 2B). It had an estimated sensitivity of 91.1% and a specificity of 80.2%; when specificity was held to 90%, the median sensitivity was estimated to be 81.0%. The GLAS algorithm was further assessed using the validation cohort data set after stratification of fibrosis and cirrhosis etiology. AUCs were comparable for viral and non-viral liver disease, with an AUC of 0.91 (95% CI: 0.86–0.96) for viral induced fibrosis/cirrhosis and 0.94 (95% CI: 0.91–0.97) for non-viral induced fibrosis/cirrhosis compared to healthy subjects. As an exploratory analysis, the GLAS algorithm was also applied to the validation cohort to discriminate patients with chronic liver disease from healthy subjects. For this application, the model had an estimated AUC of 0.65 (95% CI: 0.58–0.71), with a sensitivity of 42.9% and specificity of 80.2%.

### GLAS algorithm performance by disease state

**Fig. 3:**
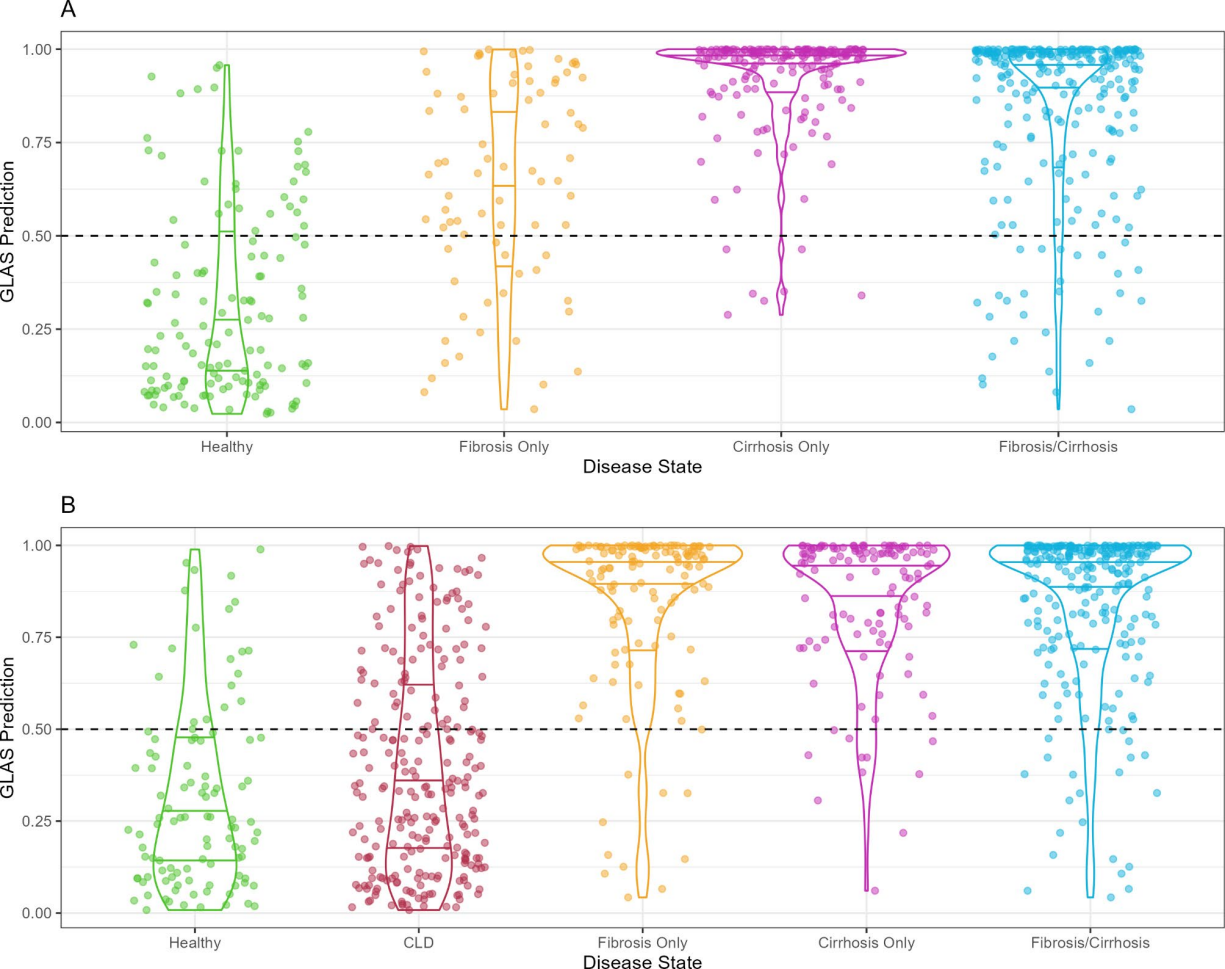
Distribution of GLAS algorithm predicted probabilities by disease state in the development **(A)** and validation **(B)** cohorts. Dashed line at 0.50 represents the cutoff for the algorithm

The performance of the GLAS algorithm was assessed by disease state in both the development and validation cohorts (Fig. 3A, B). In the development cohort, the median GLAS algorithm predicted probability was 0.959 in the fibrosis/cirrhosis group compared to 0.232 in healthy controls, and patients with cirrhosis had a median GLAS prediction of 0.983 compared to 0.671 in patients with fibrosis (Fig. 3A). In the validation cohort, the GLAS algorithm predicted probability was also higher overall in the fibrosis/cirrhosis group (median 0.949) versus healthy controls (median 0.249; Fig. 3B). In the validation cohort, the median predicted probability using the GLAS algorithm was 0.950 in patients with fibrosis versus 0.944 in patients with cirrhosis, which matches the trend observed for GP73 and LG2m biomarker concentrations in these groups (Figs. 1B and 3B). Additionally, the median GLAS prediction value for patients with chronic liver disease was 0.434, falling in between that of the healthy controls and patients with fibrosis/cirrhosis, which matches the trend seen in biomarker concentrations in each group (Figs. 1B and 3B).

## Discussion

In this pilot study, we demonstrated that serum biomarkers GP73 and LG2m, when combined with age and sex to create the GLAS algorithm, showed superior sensitivity and specificity for detection of liver fibrosis and cirrhosis. Analysis of the GLAS algorithm in an independent validation cohort showed similar clinical performance, although with lower AUC, sensitivities, and specificities. Differences in the demographics and disease classifications and etiologies between the development and validation cohorts may account for differences in biomarker levels and model performance; for example, the development cohort had a smaller number of patients with cirrhosis and a more diverse patient population than the validation cohort. Nevertheless, the GLAS algorithm remained robust for distinguishing between healthy subjects and patients with fibrosis or cirrhosis in these two highly different cohorts.

The new GLAS algorithm outperformed other algorithms used for diagnosing liver disease that are reported in the literature. In our study, Model 5 had an AUC of 0.92, sensitivity of 88.8%, and specificity of 75.9% in the development cohort and an AUC 0.93, sensitivity of 91.1%, and specificity of 80.2% in the validation cohort for detection of fibrosis or cirrhosis. These AUC values are much higher than those for the FIB-4 (AUC 0.751) and APRI algorithms (AUC 0.737) for significant fibrosis [14] (see Additional File 1). The diagnostic accuracy of the ELF algorithm, which combines detection of hyaluronic acid, type III procollagen peptide (PIIINP), and tissue inhibitor of metalloproteinase-1 (TIMP1), was evaluated in a recent meta-analysis of studies including nearly 20,000 individuals with or at risk of developing a wide variety of viral and non-viral liver diseases [35]. The analysis reported an AUC of 0.811 for detecting fibrosis, 0.812 for advanced fibrosis, and 0.810 for advanced cirrhosis. In patients with chronic HCV, the Fibrotest/FibroSure algorithm, which includes α2-macroglobulin, haptoglobin, gamma-glutamyltransferase, gamma-globulin, total bilirubin, and apolipoprotein A1, had an AUC of 0.74, sensitivity of 75.4%, and specificity of 71.4% for detection of fibrosis [36]. In a meta-analysis, the algorithm was found to have suboptimal diagnostic accuracy for fibrosis and cirrhosis in patients with HBV (AUC 0.84, sensitivity 61%, and specificity 80%) [37]. Fibrometer, which combines age, weight, platelet count, AST, ALT, ferritin, and glucose, has been evaluated for the detection of fibrosis in NAFLD. In a recent meta-analysis of 7 studies including 1616 patients with NAFLD, Van Dijk et al. reported an AUC of 0.82 (sensitivity 83.5, specificity 91.1%) for Fibrometer in detecting advanced fibrosis, and lower accuracy (0.62–0.78) for detecting significant fibrosis in 3 studies [38]. In a study of 134 patients with various autoimmune liver diseases, Fibrometer was found to have an AUC of 0.66 for severe fibrosis, which increased to 0.77 when combined with liver stiffness measured by transient elastography [39].

Differences between our findings and those of others may be related to the use of the LR model for statistical analysis, as well as differences in the patient populations. A limitation of this study was that the control groups in the development and validation cohorts were different in composition, given the retrospective nature of the analysis, which limits assessment of specific confounding variables.

This pilot study resulted in an algorithm consisting of two biomarkers (LG2m, GP73) and two demographic variables (age and sex) that demonstrated promise for predicting liver disease (fibrosis and cirrhosis). The GLAS algorithm will be further tested in cohorts with staged fibrosis and cirrhosis to evaluate its performance in early detection of liver disease. The GLAS algorithm will also be compared in head-to-head studies with other leading blood-based algorithms such as FIB4 or APRI, as well as in larger cohorts with greater geographic and racial/ethnic diversity and various liver disease etiologies.

## Conclusions

Compared to individual biomarkers, the combination of GP73 and LG2m with age and sex significantly improved the accuracy of detecting fibrosis and cirrhosis liver disease in two large and diverse patient cohorts. Further refinement of the GLAS algorithm will produce a highly accurate clinical tool to aid in the evaluation of signs of disease progression in patients with advanced liver disease. Larger longitudinal studies are needed to validate the GLAS algorithm in detecting stable versus advancing liver disease in patients over time.

### Electronic supplementary material

Below is the link to the electronic supplementary material.

Additional File 1. Current Biomarker Tests and Algorithms for Liver Disease. Description: Table listing the current biomarkers and algorithms and their performance for diagnosis of liver disease.

Additional File 2. GP73 muhuFab/CHO Conjugate Stability: 30 °C X 14 Day & 37 °C X 14 Day comparing IR (incorporation ratio of acridinium per conjugate antibody) range of conjugates. Description: Table showing conjugate stability under various temperature and time conditions.

Additional File 3. Microparticle Stability: 45 °C X 3 Days. Description: Table showing microparticle stability under various temperature and time conditions.

## Data Availability

The dataset from JHU used to develop the model is available from the corresponding author upon request. The dataset from China used to validate the model is not publicly available as it is a consortium reference set from an ongoing trial and blinding must be maintained.

## Abbreviations

AFP: Alpha fetoprotein
APRI: Aspartate platelet ratio index
AUC: Area under the curve
CLD: Chronic liver disease
ELF: Enhanced liver fibrosis
ELISA: Enzyme-linked immunosorbent assay
HAMA: Human anti-mouse antibodies
HBV: Hepatitis B virus
HCC: Hepatocellular carcinoma
HCV: Hepatitis C virus
JHU: Johns Hopkins University School of Medicine
LG2m: Laminin-gamma 2 monomer
Ln: Laminin
LoBDQ: Limit of blank, limit of detection, and limit of quantitation
LR: Logistic regression
MRE: Magnetic resonance elastography
muhuFab: Murine human chimeric Fab
PIIINP: Type III procollagen peptide
TIMP1: Tissue inhibitor of metalloproteinase-1
PIVKA-II: Protein induced by vitamin K absence/antagonist-II
PUMCH: Peking Union Medical College Hospital
ROC: Receiver operating curve
SE: Sensitivity
SP: Specificity
UTSMC: University of Texas Southwestern Medical Center
