# Supplementary material for: Validation of the novel GLAS algorithm as an aid in the detection of liver fibrosis and cirrhosis based on GP73, LG2m, age, and sex
Source: Clin Proteomics. 2023 Nov 28;20:53. doi: 10.1186/s12014-023-09444-7 (PMC10683319; doi:10.1186/s12014-023-09444-7)
Supplement: Supplementary file 1 — Additional File 1. Current Biomarker Tests and Algorithms for Liver Disease. Description: Table listing the current biomarkers and algorithms and their performance for diagnosis of liver disease. [file 12014_2023_9444_MOESM1_ESM.docx]

**Additional File 1.** Current Biomarker Tests and Algorithms for Liver Disease

| **Tests/Algorithms** | **Limitations** | **Performance** |
| --- | --- | --- |
| **Liver biopsy/pathology** | - Invasive procedure - High risk of sampling error - Risk of cancer spread along needle path | Gold standard |
| **FIB-4**  (platelet count, AST, ALT, age) | - Indirect measurement of fibrosis - Not prognostic of progression | FIB-4 AUC 0.751 vs GP73 AUC 0.898 (by HotGen ELISA) for fibrosis ≥2 [15] |
| **Enhanced liver fibrosis (ELF) test** to rule-out patients for biopsy  (hyaluronic acid, PIIINP, TIMP1) | - NICE-recommended based on Peds studies | AUC 0.811 for advanced fibrosis and cirrhosis (N=19,285 patients with considerable variability [36] |
| **Aspartate Platelet Ratio Index (APRI)**  (AST, platelet count) | - More informative for HCV than other liver diseases | APRI AUC 0.737 vs GP73 AUC 0.898 (by HotGen ELISA) for fibrosis ≥2 [15] |
| **Fibrotest (CE)/FibroSure (US)**  (α2-macroglobulin, haptoglobin, GGT, gamma-globulin, total bilirubin, apolipoprotein A1) | - Specificity issues (several biomarkers are not exclusive to liver) | AUC 0.74 for significant fibrosis in chronic HCV [37]  Suboptimal for detection of fibrosis with chronic HBV [38] |
| **Fibrometer**  (age, weight, platelet count, AST, ALT, ferritin, glucose) | - Indirect measurement of fibrosis - Poor prognosis of progression | AUCs 0.75-0.80 for advanced fibrosis in NAFLD [39]  AUC 0.81 for autoimmune liver disease [40] |
| **Fibroscan** to identify need for biopsy  (ultrasound imaging) | - Challenging in patients with ascites, obesity - High sampling error - High cost/low accessibility | AUC 0.85 for fibrosis >3 in NAFLD [41] |
| **MRE** to measure liver stiffness and assess disease stage  (magnetic resonance elastography imaging) | - High cost/low accessibility | AUC 0.841 for advanced NASH and NAFLD [42] |

AUC, area under the ROC curve; ELISA, enzyme-linked immunosorbent assay; GGT, gamma-glutamyltransferase; NAFLD, non-alcoholic fatty liver disease; NASH, non-alcoholic steatohepatitis; NICE, National Institute for Health and Care Excellence; PIINP, type III procollagen peptide; TIMP1, tissue inhibitor of metalloproteinase-1.
