# Supplementary material for: Validation of the novel GLAS algorithm as an aid in the detection of liver fibrosis and cirrhosis based on GP73, LG2m, age, and sex
Source: Clin Proteomics. 2023 Nov 28;20:53. doi: 10.1186/s12014-023-09444-7 (PMC10683319; doi:10.1186/s12014-023-09444-7)
Supplement: Supplementary file 2 — Additional File 2. GP73 muhuFab/CHO Conjugate Stability: 30 °C X 14 Day & 37 °C X 14 Day comparing IR (incorporation ratio of acridinium per conjugate antibody) range of conjugates. Description: Table showing conjugate stability under various temperature and time conditions. [file 12014_2023_9444_MOESM2_ESM.docx]

**Additional File 2.** GP73 muhuFab/CHO Conjugate Stability: 30°C X 14 Day & 37°C X 14 Day comparing IR (incorporation ratio of acridinium per conjugate antibody) for a range of conjugates. GP73 conjugates (Abbott Diagnostics China R&D) showed acceptable RLU stability with various IR with less than 20% average RLU loss.

Stability is not significantly impacted by IR.

| Test Condition | Conjugate | %Conc. Difference: Stressed vs. Non-Stressed | | | Ave Panels %Conc. Difference |
| --- | --- | --- | --- | --- | --- |
|  |  | Panel L | Panel M | Panel H |  |
| 30°C, 14DAY | IR 0.98 | -2.7% | -0.7% | 0.1% | -1.1% |
|  | IR 1.87 | -0.5% | 0.8% | -2.6% | -0.8% |
|  | IR 2.73 | -4.7% | -1.8% | -1.7% | -2.7% |
|  | IR 3.45 | -2.5% | 0.4% | -3.3% | -1.8% |
|  | IR 4.14 | -3.7% | -0.2% | 2.8% | -0.4% |
|  | IR 5.02 | -3.2% | 3.8% | -1.5% | -0.3% |
|  | IR 5.85 | -2.6% | 1.7% | -3.5% | -1.5% |
|  | LC* IR 4.12 | -5.2% | -1.5% | 0.2% | -2.2% |
| 37°C, 14DAY | IR 0.98 | -0.2% | -2.0% | 3.2% | 0.3% |
|  | IR 1.87 | 0.2% | 0.5% | -4.6% | -1.3% |
|  | IR 2.73 | -3.3% | 0.2% | -0.6% | -1.2% |
|  | IR 3.45 | -0.6% | 3.2% | 2.7% | 1.8% |
|  | IR 4.14 | -2.5% | 0.2% | 6.4% | 1.4% |
|  | IR 5.02 | -1.5% | 1.0% | 0.5% | 0.0% |
|  | IR 5.85 | -5.7% | -0.9% | 2.5% | -1.4% |
|  | LC* IR 4.12 | 0.2% | -1.9% | 1.6% | 0.0% |

*Abbott Lake County, Abbott Park, IL, USA (LC).
