# Supplementary material for: Validation of the novel GLAS algorithm as an aid in the detection of liver fibrosis and cirrhosis based on GP73, LG2m, age, and sex
Source: Clin Proteomics. 2023 Nov 28;20:53. doi: 10.1186/s12014-023-09444-7 (PMC10683319; doi:10.1186/s12014-023-09444-7)
Supplement: Supplementary file 3 — Additional File 3. Microparticle Stability: 45 °C X 3 Days. Description: Table showing microparticle stability under various temperature and time conditions. [file 12014_2023_9444_MOESM3_ESM.docx]

**Additional File 3.** Microparticle Stability: 45°C X 3 Days. GP73 IgG antibody coated on magnetic microparticles is very stable with RLU loss ≤3.5% at 45°C X 3 days for both calibrators and panels.

| Sample ID (5 reps) | Target (ng/mL) | RLU %Difference  45°C/3day vs. 2-8°C | Conc. %Difference  45°C/3day vs. 2-8°C |
| --- | --- | --- | --- |
| GP73 Cal A | 0 | -13.3% | 95.2% |
| GP73 Cal B | 3.9 | -1.4% | 3.0% |
| GP73 Cal C | 15.6 | -3.9% | 1.5% |
| GP73 Cal D | 62.5 | -2.8% | 1.7% |
| GP73 Cal E | 250 | 0.1% | 3.3% |
| GP73 Cal F | 1000 | -2.3% | -3.1% |
| Ave Cal B-F RLU% Difference | | -2.0% | 1.3% |
| Panel L | 36 | -5.9% | -1.2% |
| Panel M | 125 | -0.9% | 3.1% |
| Panel H | 500 | -3.7% | -2.5% |
| Ave Panels RLU% Difference | | -3.5% | -0.2% |
